# Supplementary material for: Expression and Variation of the Genes Involved in Rhizobium Nodulation in Red Clover
Source: Plants (Basel). 2022 Oct 28;11(21):2888. doi: 10.3390/plants11212888 (PMC9655500; doi:10.3390/plants11212888)

Supplemental Figures

Figure S1. Mapped reads from Nod+ and Nod- red clover plants to putative Nod25-like genes on Chromosome 3.
(A) *Tp3g123916815*
(B) *Tp3g123918937*
Note: Mapped reads numbers (upper right number in each column) correspond to: Nod+ Plant K10; Nod+ Plant K11; Nod+ Plant 97-5; Nod+ Plant 86-7; Nod- Plant K01; Nod- Plant 75-1; Nod- Plant 97-3; Nod- Plant 82-2; PATseq Nod+ Combined plant samples; PATseq Nod- Combined plant samples.

Figure S2. Gene structure and overall expression of the red clover NMS32/34-like genes.
(A) NMS32/34-like *Tp3g123916883*
(B) NMS32/34-like *Tp3g123916884* and *Tp3g123916889*
(C) NMS32/34-like *Tp3g123918940*
(D) NMS32/34-like *Tp3g123918941*
Note: For read tracks associated with plant samples, see note in Figure S.1.

Figure S3. Sequence alignment of the different mRNA isoforms encoding the red clover TP NMS32/34-like protein isoforms.

Figure S4. Protein Sequence alignment of the red clover NMS32/34- like gene products.

Figure S5. (A) Sequence alignment of red clover nodule-specific cysteine rich (NCR) mature peptides that contain four conserved cysteine residues.
Figure S5. (B) Sequence alignment of a few of the red clover nodule-specific cysteine rich (NCR) mature peptides that contain six conserved cysteine residues.

Figure S6. Location of the red clover embryo-specific ATS3-like/Nodule-specific PLAF domain (NPD) gene on Linkage Group 7 and mapped reads from the Nod+ and Nod- root samples.
Note: For read tracks associated with plant samples, see note in Figure S.1.

Figure S7. Protein sequence alignment of the red clover, Medicago truncatula and Arabidopsis embryo-specific ATS3-like/Nodule-specific PLAF domain (NPD) proteins.

Figure S8. Chromosome location, expression and alternate splicing of the red clover putative SYP132 (*Tp7g123897430*) and ncRNA locus (*Tp7g123897432*).
Note: For read tracks associated with plant samples, see note in Figure S.1.

Figure S1. Mapped reads from Nod+ and Nod- red clover plants to putative Nod25-like genes on Chromosome 3.

(A) Tp3g123916815

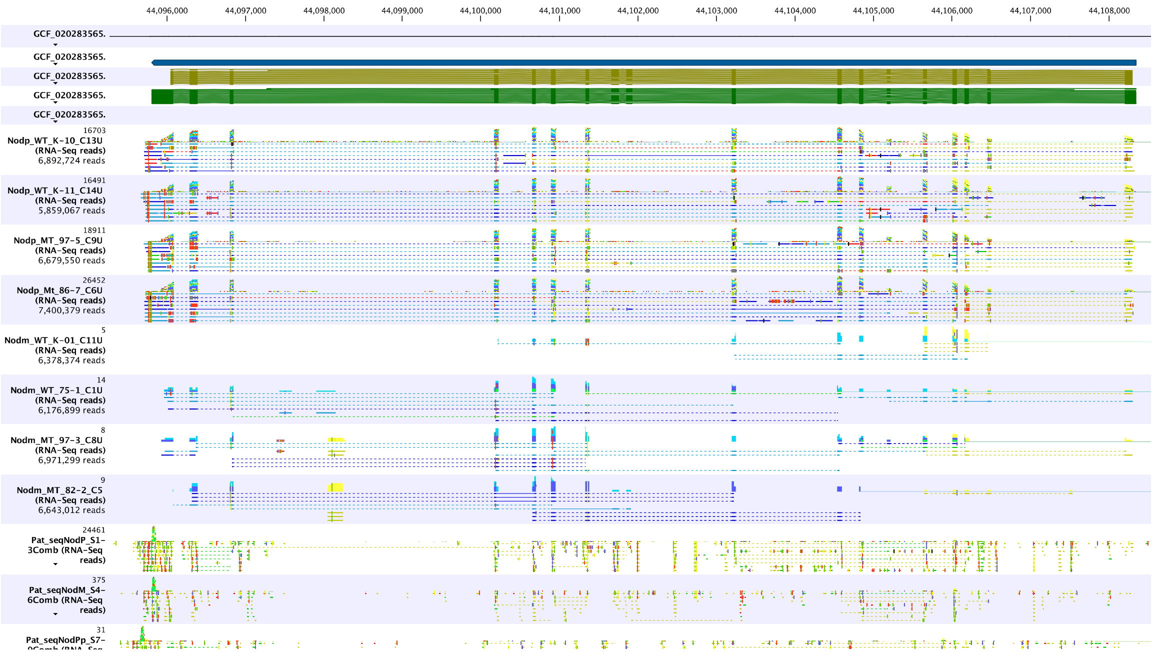

(B) Tp3g123918937

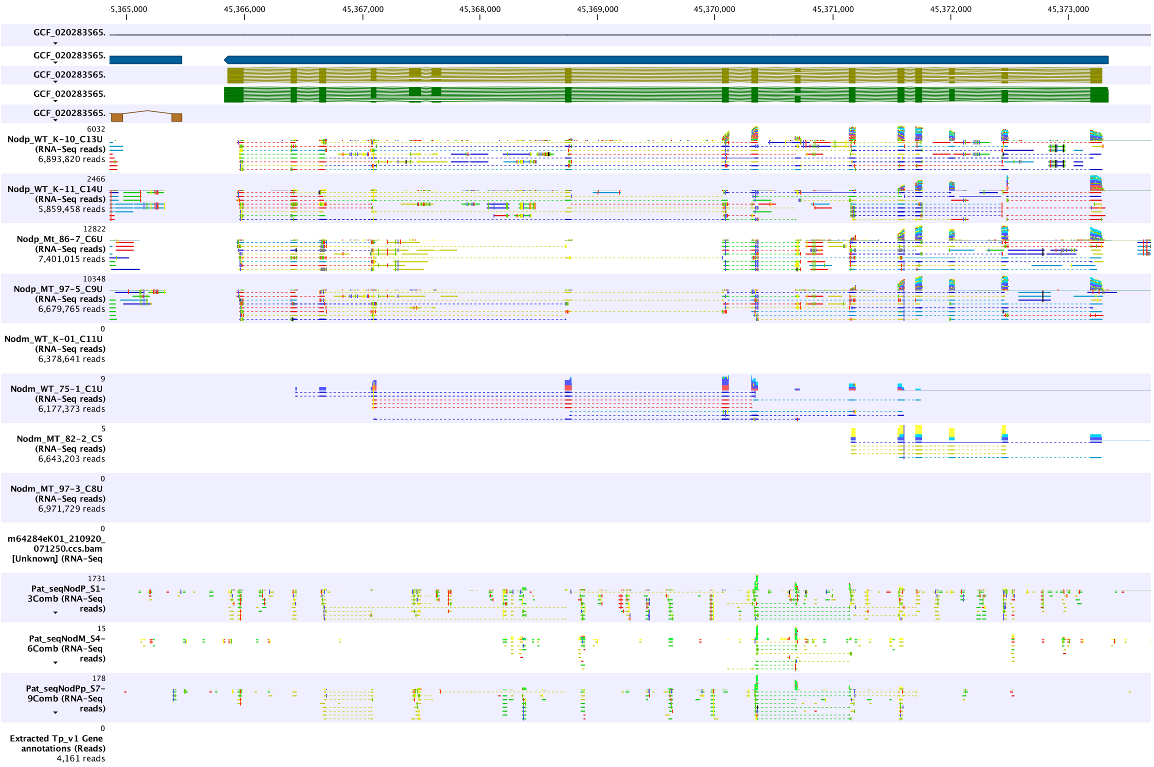

Figure S2. Gene structure and overall expression of the red clover NMS32/34-like genes.

(A) NMS32/34-like Tp3g123916883

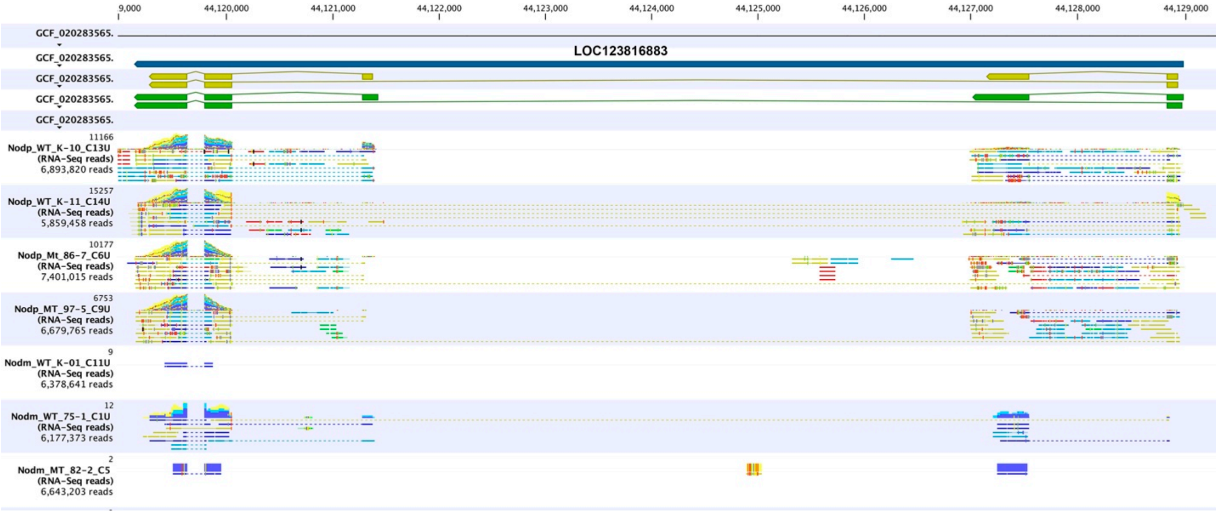

(B) NMS32/34-like Tp3g123916884 and Tp3g123916889

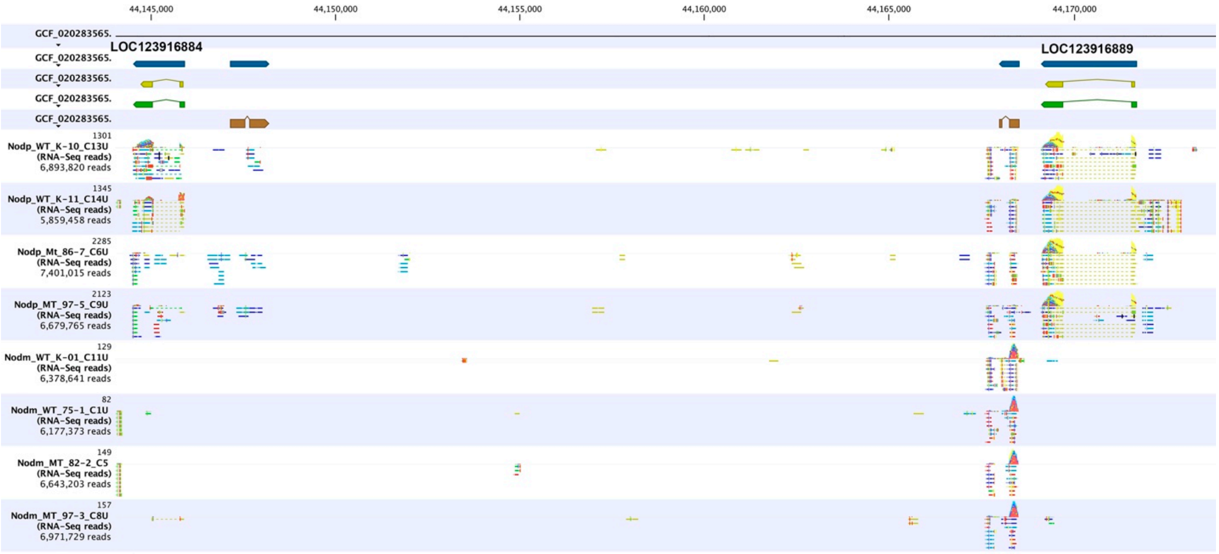

(C) NMS32/34-like Tp3g123918940

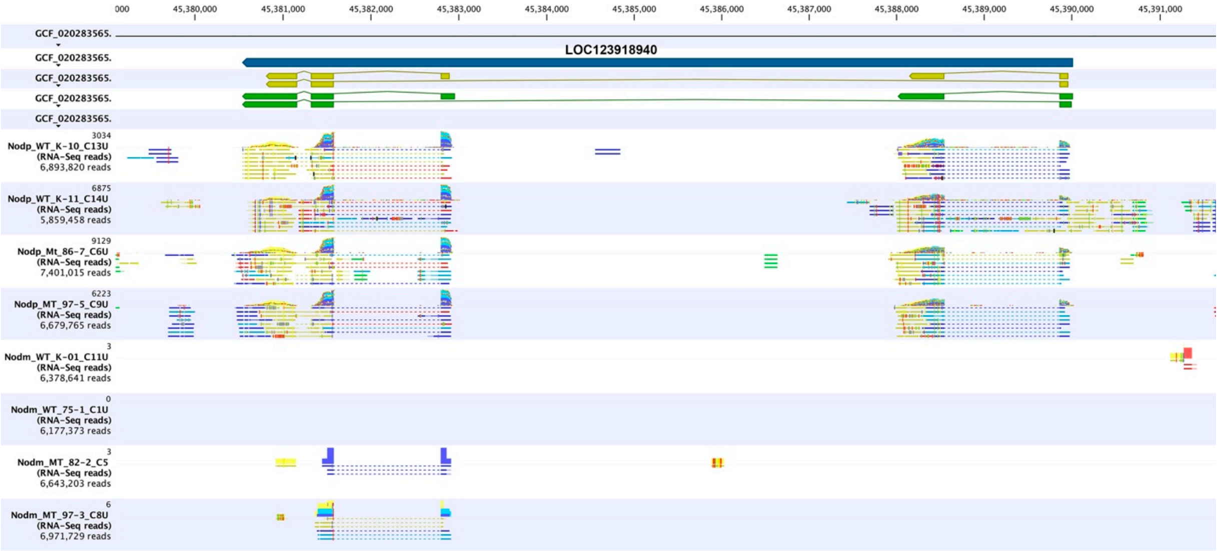

(D) NMS32/34-like Tp3g123918941

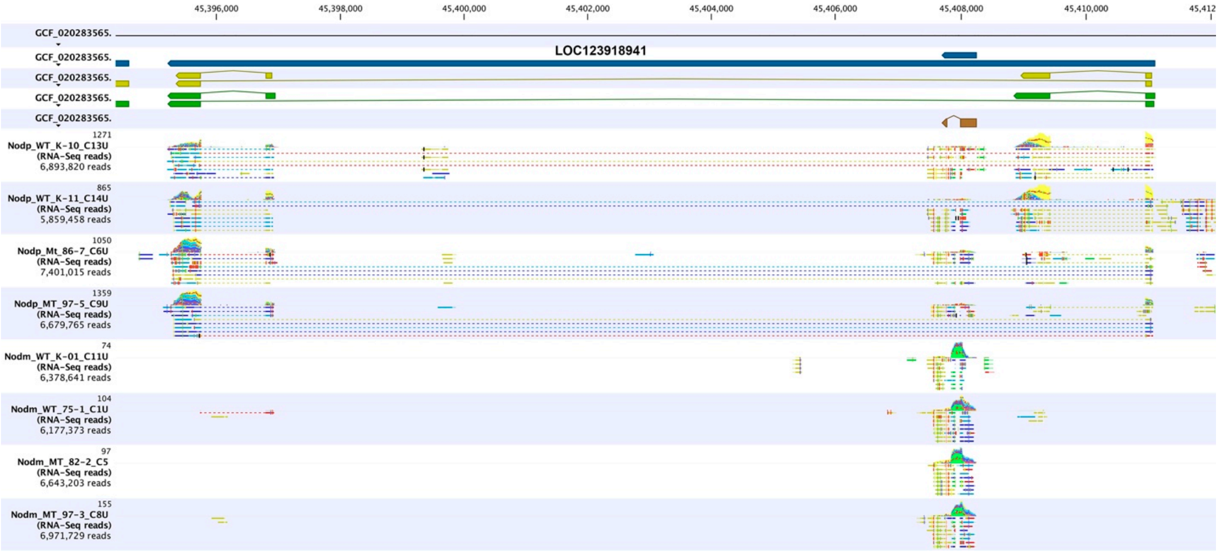

Figure S3. Sequence alignment of the different mRNA isoforms encoding the red clover TP NMS32/34-like protein isoforms.

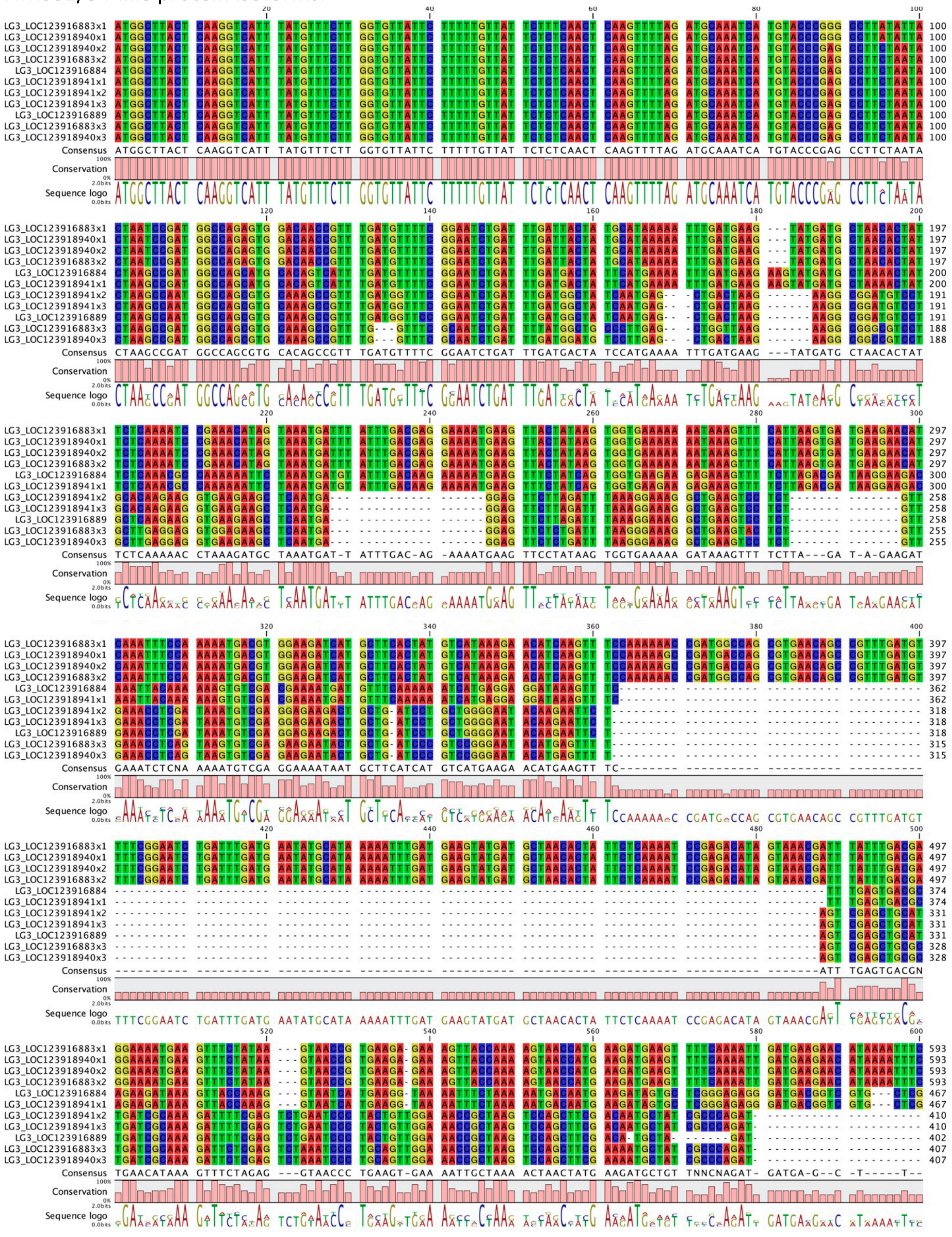

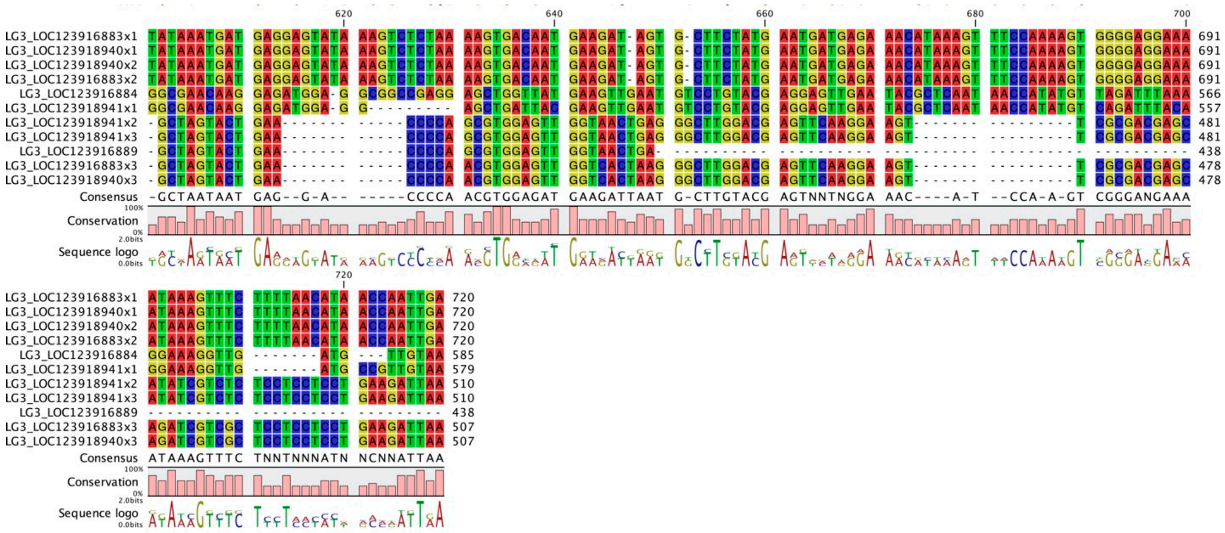

Figure S4. Protein Sequence alignment of the red clover NMS32/34- like gene products.

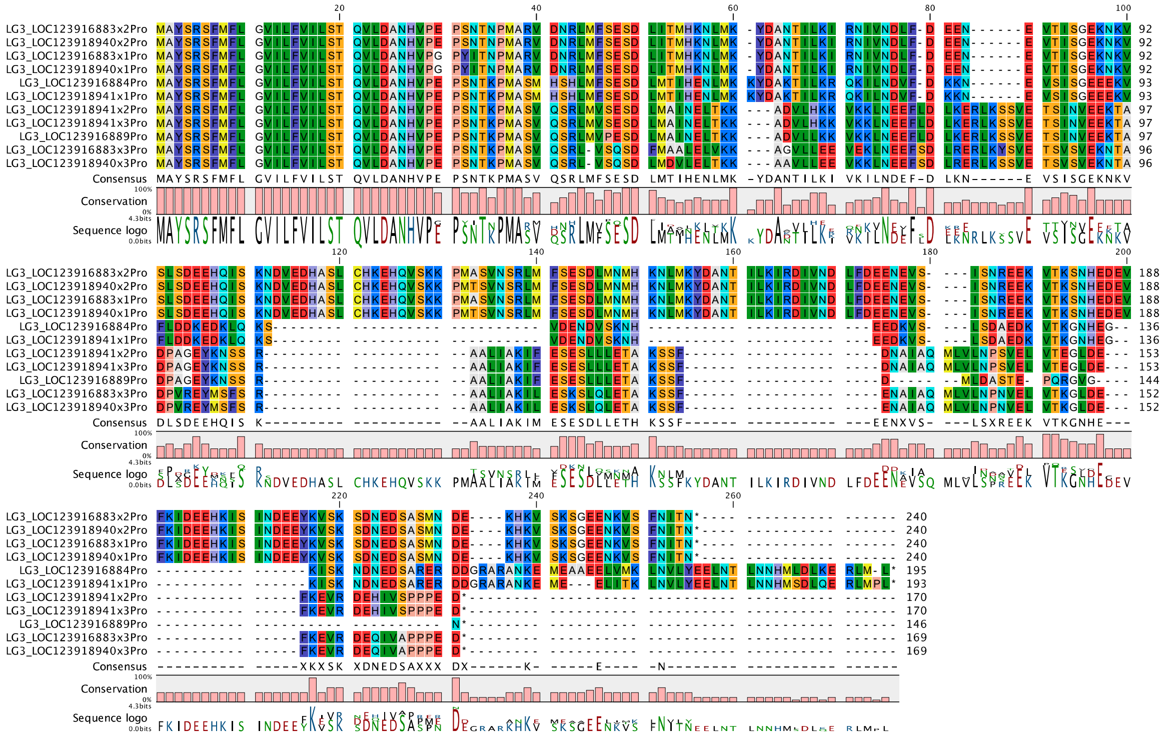

Figure S5. (A) Sequence alignment of red clover nodule-specific cysteine rich (NCR) mature peptides that contain four conserved cysteine residues.

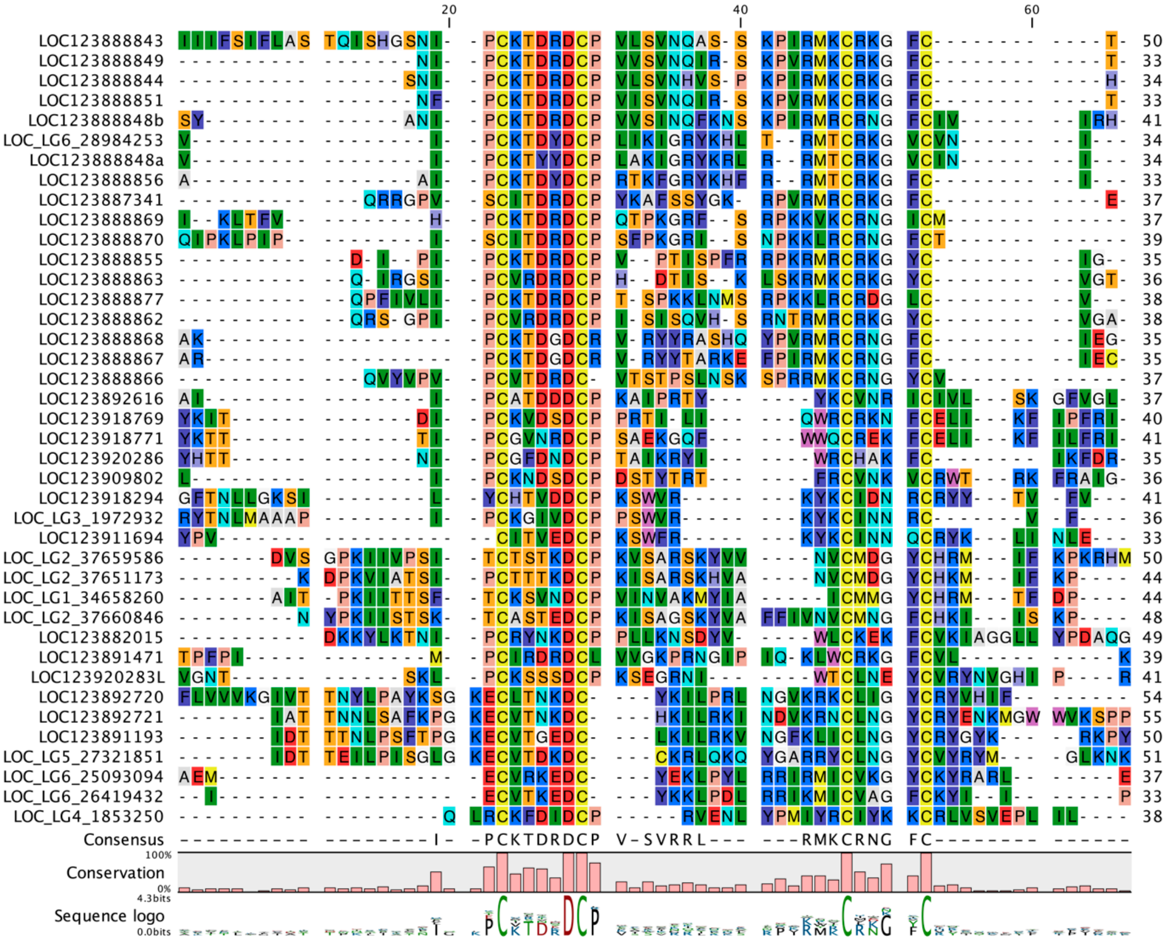

Figure S5. (B) Sequence alignment of a few of the red clover nodule-specific cysteine rich (NCR) mature peptides that contain six conserved cysteine residues.

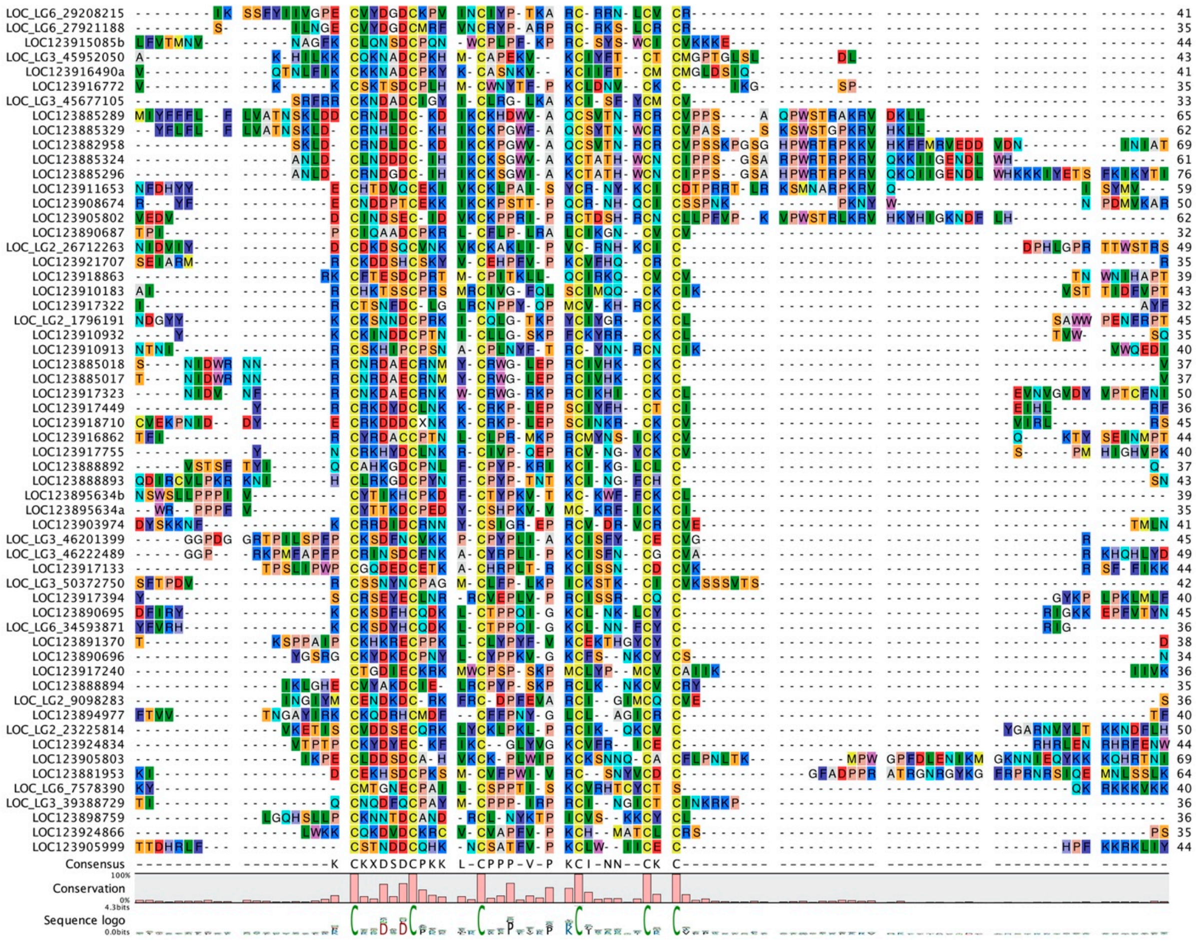

Figure S6. Location of the red clover embryo-specific ATS3-like/Nodule-specific PLAF domain (NPD) gene on Linkage Group 7 and mapped reads from the Nod+ and Nod- root samples.

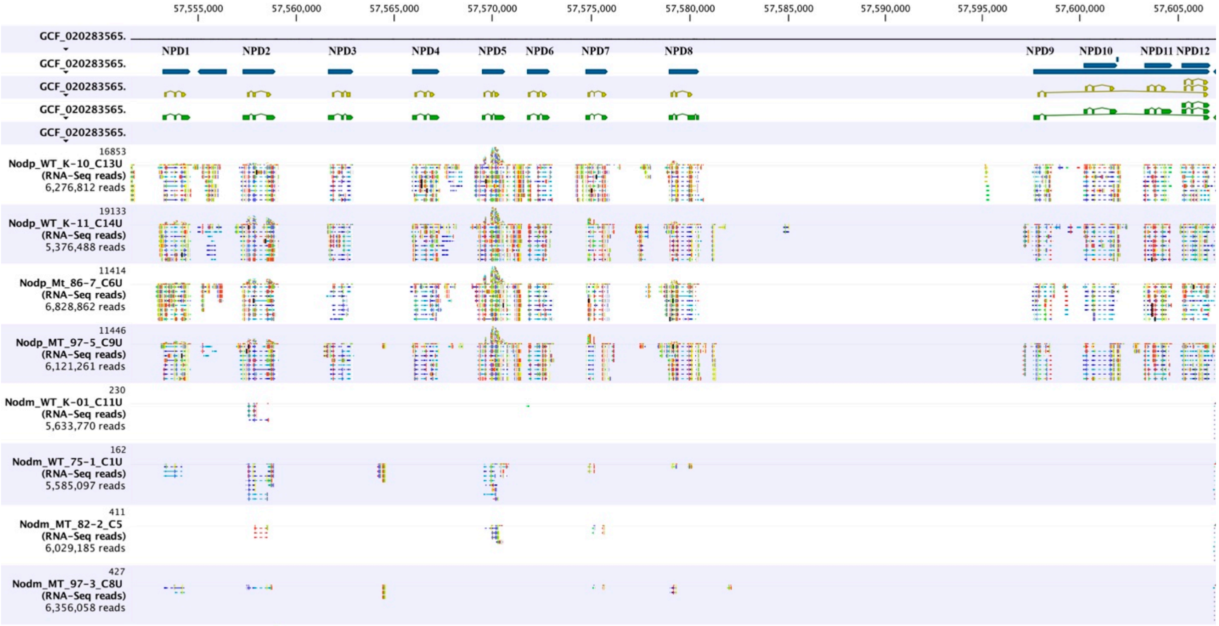

Figure S7. Protein sequence alignment of the red clover, Medicago truncatula and Arabidopsis embryo-specific ATS3-like/Nodule-specific PLAF domain (NPD) proteins.

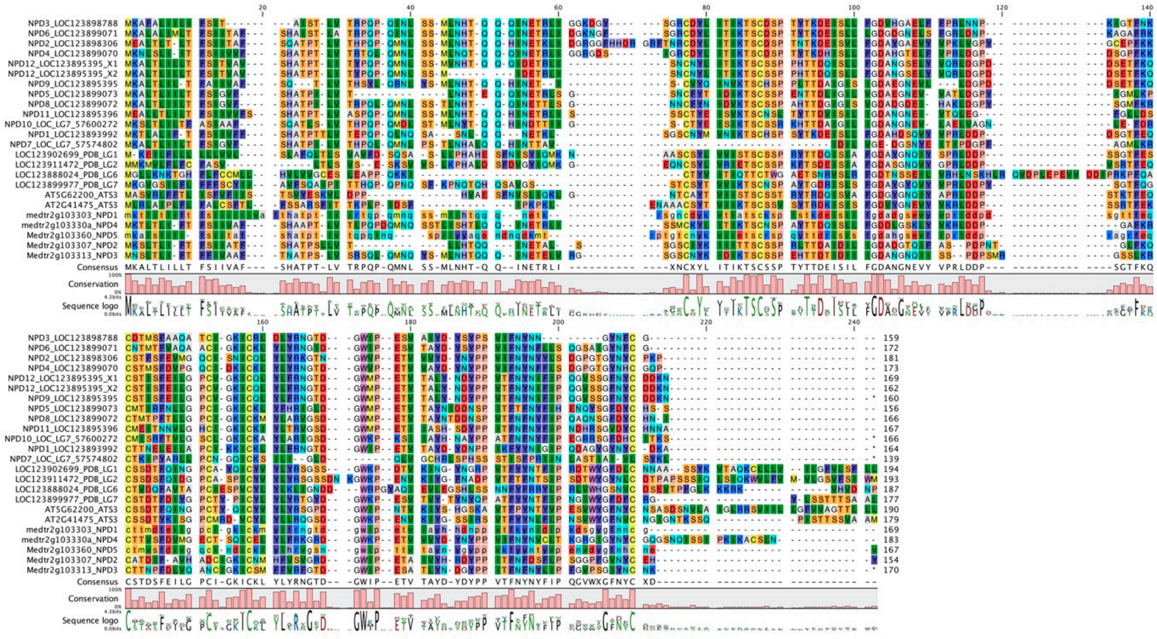

Figure S8. Chromosome location, expression and alternate splicing of the red clover putative SYP132 (*Tp7g123897430*) and ncRNA locus (*Tp7g123897432*).

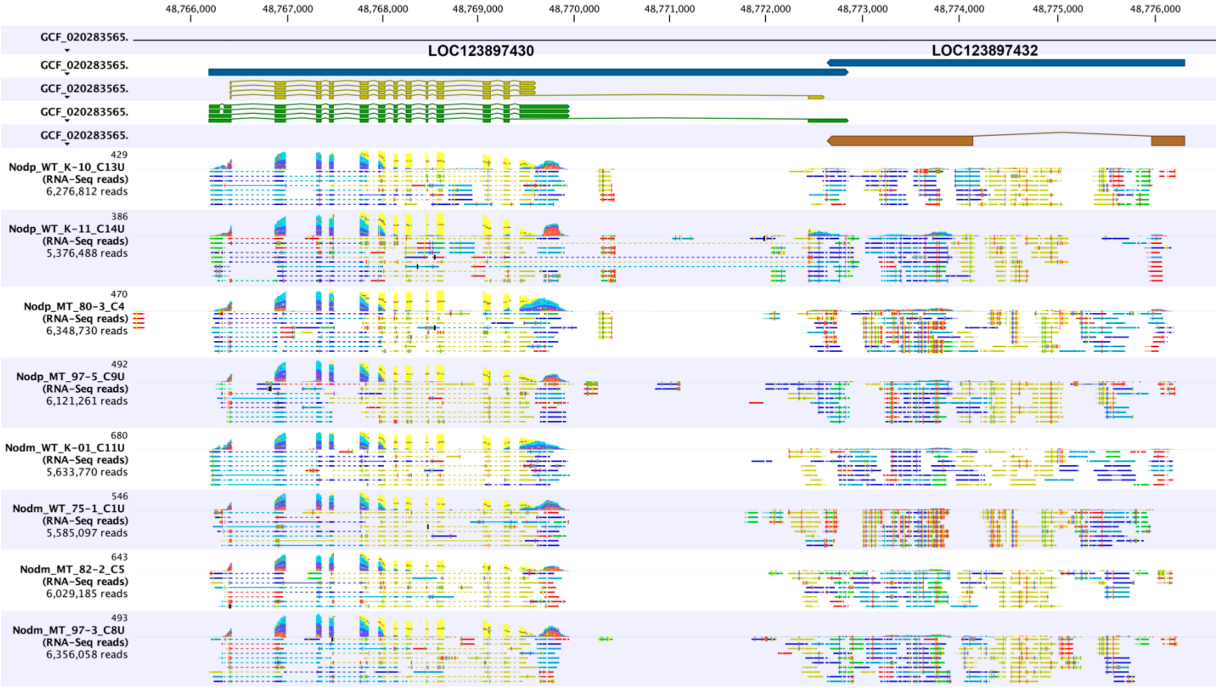

Supplement: Supplementary file 1 [file plants-11-02888-s001.zip › plants-1956248-supplementary.pdf]
